# Incidence of hip fracture among long-term care insurance beneficiaries with dementia: comparison of home care and institutional care services

**DOI:** 10.1186/s12877-019-1161-8

**Published:** 2019-05-28

**Authors:** Juyeong Kim, Young Choi, Eun-Cheol Park

**Affiliations:** 1 Department of Health & Human Performance, Sahmyook University, Seoul, Republic of Korea; 2 Department of Biomedical Informatics, Ajou University School of Medicine, Suwon, Gyeonggi-do Republic of Korea; 3 Institute of Health Services Research, Yonsei University, Seoul, Republic of Korea; 4 Department of Preventive Medicine & Institute of Health Services Research, Yonsei University College of Medicine, 50 Yonsei-ro, Seodaemun-gu, Seoul, 120-752 Republic of Korea

**Keywords:** Long-term care insurance, Hip fracture, Home care, Institutional care, Quality of care

## Abstract

**Background:**

Hip fracture among older adults is not only a major health issue but also preventable by providing proper care, but there is a lack of studies on the association between type of long-term care (LTC) service and hip fracture. This study aimed to investigate the association between the type of LTC service and the incidence of hip fracture among older adults with dementia receiving long-term care insurance (LTCI), and to investigate how such association differs according to characteristics of beneficiaries and structural characteristic of institutional care.

**Method:**

In this retrospective cohort study, data from 2008 to 2013 were collected from 7112 LTCI beneficiaries having benefit level 1 or 2 with dementia aged 60 years or over in the Korean elderly cohort data set. Type of LTC service was categorized into institutional or home care using the LTCI Claims Database, and the incidence of hip fracture was used as the outcome variable. A survival analysis using a time-dependent Cox regression analysis was performed to examine the association between time-varying LTC service type and hip fracture.

**Results:**

Of the 7112 older adults, 115 (1.6%) had hip fracture during a total of 16,540 person-years. Compared to LTC beneficiaries with home care, those with institutional care had a higher adjusted hazards ratio of incidence of hip fracture (hazards ratio = 4.33, 95% confidence interval, 2.84–6.59). This association was particularly strong among beneficiaries who did not have a danger of hip fracture during the mandatory assessment for benefit eligibility, who were partially ambulatory, who were from rural areas, and females.

**Conclusions:**

Institutional care was more likely associated with a higher incidence of hip fracture than home care. The government need to watch the institutional LTC services quality and promote improvements of the institutional care quality.

## Background

South Korea (hereafter, Korea) faces one of the fastest aging population situation in the world. Changes of social environment including lessening rates of family caregiving and lack of LTC program, made the challenges of aging population worse [1–3]. The social demand to the problem of older adults care increased in Korea. For the purpose of satisfying the care needs of older people with poor health condition and larger healthcare needs, LTCI system was implemented by the Korean government starting in July 2008 [4, 5].

Since the introduction of LTCI, the Korean government received positive reactions from public due to its role of helping elderly patients and their families [6]. LTCI mainly targets people aged 65 or over and those aged under 65 with diverse geriatric or other diseases, especially dementia, Parkinson’s disease, and stroke [7]. The payment for the LTCI system is consisted of National Health Insurance, government subsidies, and copayments [2]. Older people can receive LTC services with low financial burden of care, and they can choose the type of service between home care (HC) services and institutional care (IC) under the LTCI system, and the services are predominantly delivered in the private sector. As a result of high demand of LTC services, the number of beneficiaries increased from about 310,000 in 2010 to 387,000 in 2013 [7]. As social demands for LTCI increase, the Korean government tried to increase the quantity of LTC institutions including HC and IC. Therefore, the quantity of LTC institutions increased [7].

At the same time, the Korean government tried to improve the LTCI coverage rate. The rate of LTCI coverage rose from 1.4% in 2008 to 7.5% in 2016 [6]. Currently, the financial expense of LTCI is projected to reach 35,000 billion dollars [7]. In this situation, quantity of LTC services is enough to meet demand of LTC beneficiaries, but quality of LTC services has yet not been tested. Recently, policymakers in developed countries like U.K., Australia, and Canada are trying to improve the quality of LTC service, especially laying emphasis on improvement of measurement results of LTC services quality indicators [8]. The assessment and assurance of quality of care is important to ensure responsible health care expenses and provide safe and effective LTC services [9].

Although providing higher quality care to older adults is important given the aging population, it is not known whether quality of care is dependent on type of LTC services. Previously, validated studies have suggested hip fracture as an adequate indicator for the quality of LTC services [9]. Hip fracture is considered one of the most significant public health issues among older adults. It can have devastating consequences such as pain, immobilization, functional decline, delirium, and death. A study reported that 13% of older adults with hip fracture die within 3 months, and 24% die within 12 months [10]. Especially, elderly patient with dementia are more likely to have hip fracture due to defective neuromuscular regulation, gait apraxia, and cognitive impairment [11]. Although hip fracture is a significant concern, it is possible to prevent hip fracture by a supportive care environment. Because hip fracture typically occurs from falls when older adults lose their balance during daily movement, preventing hip fracture requires careful support, including assistance with moving and supervised daily living, which in turn requires considerable monetary and human resources [12].

Therefore, this study examined whether the type of LTC service is associated with the incidence of hip fracture among older adults diagnosed with dementia rated as levels 1 or 2 in LTCI benefit coverage level, based on a nationally representative sample of older adults population in Korea [4]. This study tested two hypotheses: (i) the type of LTC services are associated with the incidence of hip fracture, and (ii) the association between the type of LTC service and the incidence of hip fracture would differ by sex, region, whether the patient had a predetermined risk of hip fracture during the mandatory assessment of benefit eligibility, and ambulatory status.

## Methods

### Data

This study used the Korean Elderly Cohort data set from 2008 to 2013, provided by National Health Insurance Cooperation. The data set used a nationally representative sample of Korean older adults, and included LTCI claims data as well as national-level data that the Korean government requires for the clinical assessment of all LTCI beneficiaries. It includes the benefit eligibility assessment data of all enrollees and consists of the following five main sections: sociodemographic characteristics, general health status, LTC application and final decision, LTC approval checklist (LTCAC), and preliminary and adjusted entire assessment scores (LTC approval scores [LTCAS]). It also includes LTC service provider information and health insurance claims data.

The LTCI system was established to meet the high care needs of older people with poor health. Its coverage rate in the older adult population has been reported as 5.6%. Since LTC financing is limited, there is a need to confine the target population to those with high care needs of beneficiaries for maximizing the effect of LTC services and to meet the great need of LTC services. In this regard, LTC approval checklist (LTCAC) is used to distinguish those with high care need among all LTC applicants. It contains five areas a total of 69 functional assessment items including physical function (23 items, including activities of daily living [ADLs] and instrumental ADLs [IADLs]), nursing and special treatments (10 items), behavioral symptoms (16 items), cognitive function (10 items), and rehabilitation needs (10 items). The LTCAC is measured by employees from NHI. Subsequently, the Need Assessment Committee calculate the preliminary LTCAS scores for 59 items based on LTCAC (excepting IADLs) following national guidelines by using a complex, highly nonlinear formula. Finally, the committee issues a final LTCI benefit coverage level from 1 to 3 after considering individual’s situations and needs of special service and a physician’s opinion. Lower levels indicate bigger dependency and a higher benefit amount (Level 1: very severe, Level 2: severe, Level 3: moderate). The national LTCI assessment, which was conducted initially in 2008 to identify the number of potential beneficiaries and determine the severity of their condition, served as the baseline assessment for all beneficiaries in the data set. Specific guidelines for the assessment and documentation ensure the quality of the data set.

A mandatory assessment for LTCI benefit eligibility is conduct every year, except for those older adults with an initial high score (> 100). A team consisting of nurses or social workers from LTCI fulfills the assessments to judge the LTC needs of older adults, using first hand observations of beneficiaries and interviews with families or LTC workers. Examiners conduct assessments including based on the LTCAC manual.

### Study population

The eligibility criteria for the present study were a main diagnosis of dementia and a LTCI benefit coverage level of 1 or 2. These criteria were considered the most convincing for determining the need for LTC services. Those with benefit level of 1 or 2 have the option of choosing between HC and IC services. The exclusion criteria were individuals with a benefit level of 3, receiving HC services only in special exceptions and not having comparable characteristics to other participants in the HC and IC groups [13]. Additionally, individuals diagnosed with hip fracture prior to receiving LTCI benefits were excluded. Among the 99,841 LTCI beneficiaries in July 2008, 35,421 who had an LTCI benefit coverage level of 1 or 2 were selected. Of those, 10,491 had dementia. After excluding those who did not use LTC services and those diagnosed with hip fracture before becoming LTCI beneficiaries, 7112 beneficiaries were included in the final dataset.

### Study variables

#### Type of long-term care service

HC and IC were considered as LTC services in this study, excluding the special cash type. HC includes home bathing, home help, adult day and night care centers, skilled nursing services, and medical equipment rental services (e.g., in-tub bath lifts, wheelchairs, and specialty care mattresses). It enable beneficiaries to use various types of HC services within a monthly funding limit [14]. IC contains social and recreation therapy, 24-h nursing care, rehabilitation, and other conveniences [13].

#### Hip fracture

Hip fracture was defined as a record indicating a diagnosis of femoral fracture. Specifically, we retrieved all claims records for the hospital admissions of patients aged 60 years or older diagnosed with femoral fracture (based on International Classification of Diseases, Tenth Edition [ICD-10] diagnostic codes: S72). The diagnosis codes were selected based on previous studies [15].

We intended to identify the association between the incidence of hip fracture and LTC service type. Therefore, we defined the incidence of hip fracture by LTC service type as the occurrence of a diagnosis of hip fracture during the period between the start and end dates of a given LTC service.

### Control variables

Socio-demographic variables included age (60–74, ≤ 80, ≤ 85, ≤ 90, or > 90 years), sex (male/ female), region (urban/rural), equivalent household income (high, middle, or low), primary caregiver (spouse, children, care assistant, or none), cohabitation (living alone, living with the spouse, family members, caregivers from long term care facilities, or others), and year. Information on socio-demographic variables was collected from corresponding section of the LTCI benefit eligibility assessment data. Health-related variables included predetermined risk of fracture on the during the mandatory assessment for benefit eligibility was conducted (yes/ no), ambulatory status (possible, partially possible, or impossible), LTCI benefit coverage level (1/2), Charlson Comorbidity Index (CCI; 0, 1, 2, or ≥ 3) [16], LTCAS score, ADL score, cognitive function score, and behavioral symptoms score. Information on these variables were collected from the LTCAC findings in the LTCI benefit eligibility assessment data. Information about socio-demographic and health-related variables was obtained since the year prior to the commencement of LTCI services. Only the comorbidity component of the CCI was calculated, and all diagnostic information was collected from inpatient and outpatient billing data within the diagnosis year.

For structural characteristics, ownership type of LTC service providers (local government, non-profit, or private) and the number of clinic room in the IC facility (with or without a clinic room) were included. This information was collected by matching an LTC provider’s ID with LTC providers’ information and LTCI claim data.

### Statistical analysis

Descriptive statistics were conducted for all variables as frequencies and percentages for categorical variables using the Chi-squared test. The survival probability for the incidence of hip fracture was estimated by the Kaplan–Meier product limit method with log-rank tests to stratify type of LTC services. In order to investigate the association between type of LTC services and the incidence of hip fracture, we analyzed the cumulative mean function estimate for recurrent events data using a Cox’s proportional hazards model [17]. The log of cumulative hazards was proportional to the follow-up time, and no violations of the proportional hazards assumption were detected. Subgroup analyses were conducted to examine the association between type of LTC services and the incidence of hip fracture by sex, region, having a predetermined risk of fracture on the mandatory assessment for determining LTC benefit eligibility. Additionally, subgroup analyses were conducted to identify the association between structural characteristics (number of clinic room) of IC facilities and incidence of hip fracture among beneficiaries receiving IC as compared to those receiving HC. All statistical analyses were performed using SAS statistical software version 9.4 (SAS Institute, Cary, NC, USA).

## Results

Table 1 shows the frequencies and percentages for all variables at baseline, stratified by censored status. Of the 7112 eligible participants, 115 (1.6%) experienced hip fracture during the study period. At baseline, 39.4% of the study population received IC, and 60.6% received HC.

**Table 1:** Baseline Characteristics by Incidence of Hip Fracture

Variable	N	Col %^a^	No Hip Fracture		Hip Fracture		P-value
			N	Row %^b^	N	Row %^b^	
Total	7112	100.0	6997	98.4	115	1.6	
Type of care							
Institutional care	2802	39.4	2712	96.8	90	3.2	<.0001
Home care	4310	60.6	4285	99.4	25	0.6	
Age							
60–74	1131	15.9	1117	98.8	14	1.2	0.380
75–80	1795	25.2	1769	98.6	26	1.5	
81–85	1810	25.5	1782	98.5	28	1.6	
86–90	1454	20.4	1428	98.2	26	1.8	
91≤	922	13.0	901	97.7	21	2.3	
Sex							
Male	1963	27.6	1951	99.4	12	0.6	<.0001
Female	5149	72.4	5046	98.0	103	2.0	
Region							
Urban	2835	39.9	2791	98.5	44	1.6	0.724
Rural	4277	60.1	4206	98.3	71	1.7	
Income level							
Low	2565	36.1	2515	98.1	50	2.0	0.170
Middle	1173	16.5	1153	98.3	20	1.7	
High	3374	47.4	3329	98.7	45	1.3	
Primary Caregiver							
Spouse	1277	18.0	1271	99.5	6	0.5	<.0001
Children	3285	46.2	3243	98.7	42	1.3	
Care assistant	2341	32.9	2280	97.4	61	2.6	
None	209	2.9	203	97.1	6	2.9	
Cohabitant							
Living alone	360	5.1	358	99.4	2	0.6	<.0001
Spouse (one generation family)	1096	15.4	1088	99.3	8	0.7	
Family members (two generation family)	3127	44.0	3083	98.6	44	1.4	
Caregivers from Long term care facilities	1593	22.4	1548	97.2	45	2.8	
Others	936	13.2	920	98.3	16	1.7	
Having danger of fracture based on the mandatory assessment for long term care benefit^c^							
Yes	5457	76.7	5379	98.6	78	1.4	0.023
No	1655	23.3	1618	97.8	37	2.2	
LTCI benefit coverage level							
1	183	2.6	179	97.8	4	2.2	0.537
2	6929	97.4	6818	98.4	111	1.6	
Charlson Comorbidity Index (CCI)							
0	1655	23.3	1630	98.5	25	1.5	0.738
1	2167	30.5	2136	98.6	31	1.4	
2	1540	21.7	1513	98.3	27	1.8	
3+	1750	24.6	1718	98.2	32	1.8	
LTC approval score (LTCAS, mean ± SD)			81.5 ± 8.1		83.8 ± 13.0		
ADL (mean ± SD)			32.3 ± 4.1		33.6 ± 5.2		
Cognitive function score (mean ± SD)			7.0 ± 2.1		7.0 ± 1.9		
Behavioral symptoms score (mean ± SD)			3.6 ± 3.2		3.3 ± 3.1		
Ambulatory status							
Ambulatory	1243	17.5	1225	98.6	18	1.5	0.003
Partially ambulatory	2800	39.4	2770	98.9	30	1.1	
Not ambulatory	3069	43.2	3002	97.8	67	2.2	
Ownership type							
Local government	277	3.9	269	97.1	8	2.9	0.077
Non-profit	2836	39.9	2784	98.2	52	1.8	
Private	3999	56.2	3944	98.6	55	1.4	

^a^It presented column percentage. Column percentage shows the proportion of a total of 7112 participants in each category

^b^It presented row percentage. Row percentage add up to each row total, and shows the proportion within each characteristics in each row by whether having the incidence of hip fracture or not

^c^The record regarding having pressure ulcer was based on the information of patients’ mandatory assessment data, which is requested to qualify to become a long-term care beneficiaries

Regarding the association between the LTC service type and the incidence of hip fracture, 90 (3.2%) of the 2802 participants who received IC experienced hip fracture during the follow-up period, compared to only 25 (0.6%) of the 4310 participants who received HC. We observed significant differences in the incidence of hip fracture by type of care, sex, primary caregiver, cohabitant, having a predetermined risk of fracture based on the mandatory assessment for determining LTC benefit eligibility, and ambulatory status.

**Fig. 1:**
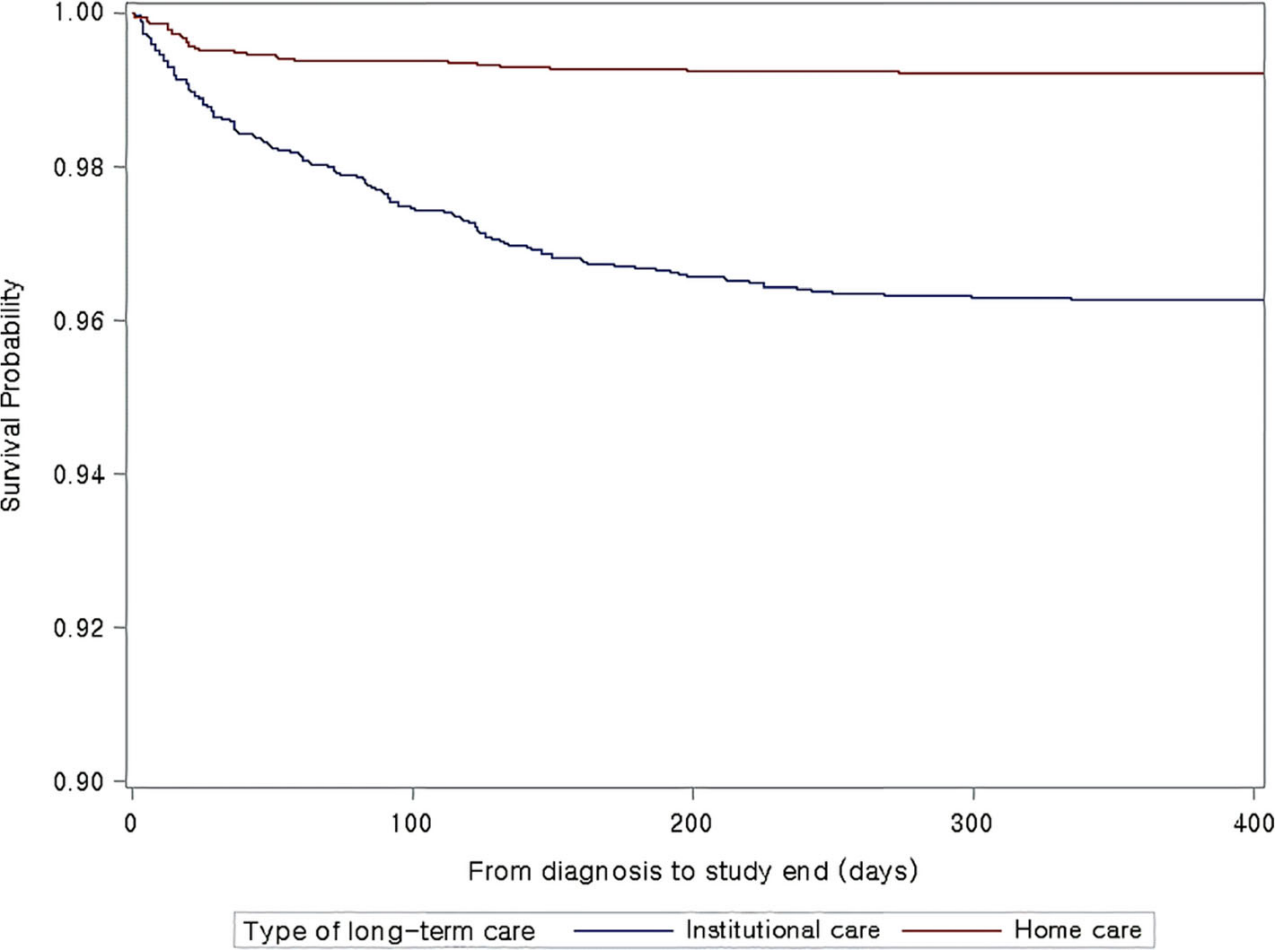
The mean survival time of those with home care and institutional care was 325.5 days and 271.3 days, respectively. The difference in survival for the incidence of hip fracture in participants was greatest at 400 days (*p*-value < 0.0001 by log-rank test). The survival probability for the incidence of hip fracture in those older adults with IC is more likely to rapidly decrease over time than the survival probability of those older adults with HC

Figure 1 shows the result of Kaplan–Meier analysis. The mean survival time of those receiving HC and IC was 325.5 days and 271.3 days, respectively. The difference in survival for the incidence of hip fracture in participants was greatest at 400 days (*p*-value < 0.0001 by log-rank test).

Table 2 shows the result of the Cox proportional hazards regression analysis of the adjusted effect of the type of LTC service on the incidence of hip fracture. As compared to those receiving HC, those receiving institutional care had a higher adjusted hazards ratio (HR) for hip fracture (HR = 4.23, 95% CI, 2.83–6.58).

**Table 2:** Adjusted hazard ratios for factors associated with hip fracture

Variable	Hip fracture		
Total	HR^b^	95% CI	
Type of care			
Institutional care	4.32	2.83	6.58
Home care	1.00		
Age			
60–74	1.00		
75–80	1.18	0.71	1.95
81–85	1.30	0.78	2.16
86–90	1.40	0.82	2.37
91≤	2.30	1.33	3.98
Sex			
Male	0.79	0.51	1.23
Female	1.00		
Region			
Urban	1.16	0.86	1.57
Rural	1.00		
Income level			
Low	1.00		
Middle	0.90	0.57	1.41
High	0.95	0.69	1.31
Primary Caregiver			
Spouse	0.21	0.07	0.67
Children	0.47	0.23	0.96
Care assistant	1.00		
None	0.34	0.18	0.65
Cohabitant			
Living alone	1.00		
Spouse (one generation family)	2.54	0.73	8.90
Family members (two generation family)	2.16	0.83	5.63
Caregivers from Long term care facilities	2.98	1.17	7.60
Others	1.53	0.57	4.09
Having danger of fracture based on the mandatory assessment for long term care benefit^a^			
Yes	1.00		
No	1.28	0.91	1.80
LTCI benefit coverage level			
1	0.44	0.15	1.32
2	1.00		
Charlson Comorbidity Index (CCI)			
0	1.00		
1	1.02	0.67	1.56
2	1.37	0.88	2.15
3+	1.86	1.21	2.87
LTC approval score (LTCAS, mean ± SD)	1.00	0.98	1.03
ADL (mean ± SD)	1.04	0.99	1.09
Cognitive function score (mean ± SD)	0.98	0.91	1.05
Behavioral symptoms score (mean ± SD)	0.92	0.86	0.98
Ambulatory status			
Ambulatory	1.00		
Partially ambulatory	1.12	0.67	1.87
Not ambulatory	1.96	1.19	3.22
Ownership type			
Local government	1.00		
Non-profit	0.72	0.42	1.26
Private	0.73	0.41	1.29

^a^The record regarding having danger of fracture was based on the information of patients’ mandatory assessment data, which is requested to qualify to become long-term care beneficiaries

^b^Adjusted for age, sex, region, income level, primary caregiver, cohabitant, having danger of fracture based on the mandatory assessment for long-term care benefit, LTCI benefit coverage level, CCI, LTCAS, ADL, cognitive function score, ambulatory status, and ownership type

Table 3 outlines the findings of the subgroup analysis according to gender, income level, region, predetermined risk of fracture on the mandatory assessment for determining LTC benefit eligibility, and ambulatory status. Among those receiving IC, the following groups all had a higher adjusted HR for hip fracture: participants who were female (HR = 4.73, 95% CI, 2.96–7.53 vs. male: HR = 3.54, 95% CI, 1.24–10.09), those residing in rural areas (HR = 4.69, 95% CI, 2.60–8.44 vs. urban areas: HR = 4.26, 95% CI, 2.27–7.90), those who did not have a predetermined risk of fracture based on the mandatory assessment for determining LTC benefit eligibility (HR = 5.27, 95% CI, 2.15–12.92 vs. those who had a predetermined risk: HR = 4.09, 95% CI, 2.53–6.62), and those who were partially ambulatory (HR = 4.96, 95% CI, 2.24–11.00) and not ambulatory (HR = 4.62, 95% CI, 2.69–7.97 vs. those who were ambulatory: HR = 2.21, 95% CI, 0.61–8.02).

**Table 3:** Result of subgroup analysis regarding the association between type of care and the incidence of hip fracture by factors

Variable	Type of care					
	Institutional care			Home care		
Total	HR^b^	95% CI		HR^b^	95% CI	
Sex						
Male	3.54	1.24	10.09	1.00		
Female	4.73	2.96	7.53	1.00		
Region						
Urban	4.26	2.27	7.90	1.00		
Rural	4.69	2.60	8.44	1.00		
Having danger of fracture based on the mandatory assessment for long term care benefit^a^						
Yes	4.09	2.53	6.62	1.00		
No	5.27	2.15	12.92	1.00		
Ambulatory status						
Ambulatory	2.21	0.61	8.02	1.00		
Partially ambulatory	4.96	2.24	11.00	1.00		
Not ambulatory	4.62	2.69	7.94	1.00		

^a^The record regarding having danger of fracture was based on the information of patients’ mandatory assessment data, which is requested to qualify to become long-term care beneficiaries

^b^Adjusted for age, sex, region, income level, primary caregiver, cohabitant, having danger of fracture based on the mandatory assessment for long-term care benefit, LTCI benefit coverage level, CCI, LTCAS, ADL, cognitive function score, ambulatory status, and ownership type

Table 4 outlines the findings of the subgroup analysis of the association between structural characteristics of the institution and incidence of hip fracture as compared with beneficiaries with HC. As compared to those received HC, participants who received IC without a clinic room had a higher adjusted HR for hip fracture (HR = 4.55, 95% CI, 2.69–7.70).

**Table 4:** Adjusted hazard ratios regarding the association between structural characteristics of institution and incidence of hip fracture among beneficiaries with institutional care

Variable		Hip fracture		
		HR^a^	95% CI	
Number of clinic room				
Home care		1.00		
Institutional care	without clinic room	4.55	2.69	7.70
	with clinic room	4.26	2.77	6.57

^a^Adjusted for age, sex, region, income level, primary caregiver, cohabitant, having danger of fracture based on the mandatory assessment for long-term care benefit, LTCI benefit coverage level, CCI, LTCAS, ADL, cognitive function score, ambulatory status, and ownership type

## Discussion

This study examined the relationship between type of LTC services and incidence of hip fracture among elderly with LTCI benefit coverage level 1 or 2 with dementia using nationwide elderly cohort data. According to the results controlling for potential confounders, IC was found to be associated with a higher incidence of hip fracture compared to HC. In particular, the association between type to LTC services and the incidence of hip fracture was especially high among IC beneficiaries who were female, lived in rural areas, had no predetermined risk of fracture based on the mandatory assessment for LTC benefits, and who were partially able or unable to ambulate.

In comparison with previous studies, there is a one study examined the association between type of LTC and incidence of pressure ulcer in Korean elderly patients with dementia; although that study used development of pressure ulcers instead of hip fracture as a quality indicator in LTC, the results are comparable to our study and found that beneficiaries with IC were more likely to be associated with the incidence of pressure ulcers compared to those with HC [18]. Another study tested whether the type of LTC services is associated with cognitive function, physical functioning, behavioral symptoms. In that study, IC users were associated with poor cognitive function, poor physical functioning, and better behavioral symptoms compared with those receiving HC services [13].

In this study, older adults with IC services were found more likely to be associated with the incidence of hip fracture than HC users, and this result can be explained by the different care environment. Older adults receiving HC services are able to obtain care from their family caregiver when any LTC worker is not around. On the other hand, those who receive IC services live in a LTC institution for 24 h, and they are in a situation in which they have to fully depend of LTC workers for every care need [19]. To prevent hip fracture, proper care and supervision from a daily caregiver is important. Also, living in an environment with the use of protective equipment to prevent hip fracture is important [12], and providing this environment is one role of LTC services. A previous study found that fall-related knowledge among nursing home workers is not adequate to prevent accidental falls, and therefore, re-education is needed for fall prevention [20]. Also, previous studies have shown that patients who live with family members who know how to prevent hip fracture have a lower risk of fall [21]. The finding of this study shows that although patients in institutional care facilities are fully dependent on LTC workers, these workers are providing inadequate preventive care for hip fractures. Another previous study showed that elderly adults in institutional care tend to have poorer health than those who receive home care [22]. For these reasons, beneficiaries who receive institutional care may be at a higher risk for hip fracture.

In the subgroup analysis by region, the effect of receiving institutional care on the incidence of hip fracture was larger among those who lived in rural areas compared to urban areas. This can be explained by the regional inequality of care services and different characteristics between urban and rural residences. Previous studies have reported that rural nursing home facilities were associated with poorer quality of care [23]. Also, rural residents were more likely to be older and poor, with a worse health status, and less likely to receive preventive services and visit health care providers [24].

In the subgroup analysis by gender, the effect of receiving institutional care on the risk of hip fracture was greater among females than males. A previous study found that older female adults were more vulnerable to falls [25]; therefore, older female adults should receive more focused care to prevent falls. The results of the present study suggest that proper care was not fully provided from LTC workers to older female adults, who are more likely to have fall injuries.

The subgroup analysis indicated that incidence of hip fracture was particularly high among beneficiaries who had no predetermined risk of fracture during their mandatory assessment for benefit eligibility. Proper care and a living environment that prevents falls in daily life are essential for reducing fall injuries including hip fracture among all elderly people, considering their frailty and low mobility [12]. However, LTC workers are likely to focus their daily attention on patients with a known a predetermined risk of fracture, rather than those with no known risk. This naturally deprioritizes preventative care of hip fracture for beneficiaries without a predetermined risk of fracture based on the mandatory assessment. Similar reasoning might explain why the risk of hip fracture was particularly high among those who were not only non-ambulatory but also partially ambulatory: those who are not ambulatory are a high-risk population, and thus receive focused care for preventing hip fracture, whereas those who are ambulatory have a low risk of hip fracture.

This study has several strengths. To the best of our knowledge, this is the first study to examine the associations between type of LTC services and incidence of hip fracture based on nationally representative sample.

This study has some limitations as well. Firstly, the incidence of hip fracture could be underestimated. Medical practitioners could under-report the incidence of hip fracture on medical records to report better quality indicator of LTC services. Second, the reason for higher incidence of hip fracture among older persons living in facility could be due to a longer distance to fulfill daily activities (e.g., from bedroom to bathroom or to living room) but the factors regarding this issue such as average walking time or the way of living was not able to consider in this study due to data constraint. In addition, the observational nature of our study leaves room for residual confounding and other potential sources of bias.

This study has several implications. First, the government need to watch the institutional LTC services quality and promote improvements of the IC services quality. To promote IC services quality improvement, payments system to LTC providers should be reformed to encourage LTC provider to improve services quality. For example, the results of service quality assessment in the process of payment. Second, LTC workers should be careful not to disregard preventative care of hip fracture for those older adults without a predetermined risk of fracture during mandatory assessment for benefit eligibility, who are unable to ambulate and who are partially ambulatory, and who are female. Finally, efforts are needed to increase LTC service quality for beneficiaries living in rural area. Since the introduction of LTC in 2008, efforts to increase the quantity of long-term care across Korea have been made, but efforts to alleviate the differences in quality of LTC services between urban and rural areas have been inadequate. Considering that large number of older people live in rural areas, efforts should be made to eliminate regional differences in quality of LTC services.

## Conclusion

LTC beneficiaries who receive institutional care had higher adjusted HRs of hip fracture than did those who received home care. The government should monitor and promote LTC service providers to improve the quality of IC.

## Data Availability

Researchers interested in using Korean Elderly Cohort data may access the data from the following sites: https://nhiss.nhis.or.kr/bd/ab/bdabd003cv.do. The authors do not have the legal right to distribute or provide access to the data set.

## Abbreviations

ADL: Activities of daily living
CCI: Charlson Comorbidity Index
HC: Home care
HR: Hazards ratio
IADL: Instrumental ADLs
IC: Institutional care
ICD-10: International Classification of Diseases, Tenth Edition
LTC: Long-term care
LTCAC: LTC checklist
LTCAS: LTC approval scores
LTCI: Long-term care insurance
